# Academic Stress and Self-Regulation among University Students in Malaysia: Mediator Role of Mindfulness

**DOI:** 10.3390/bs8010012

**Published:** 2018-01-15

**Authors:** Nur Hamizah Hj Ramli, Masoumeh Alavi, Seyed Abolghasem Mehrinezhad, Atefeh Ahmadi

**Affiliations:** 1 Psychology Department, Faculty of Social Sciences and Liberal Arts, UCSI University, Kuala Lumpur 56000, Malaysia; Meez.Hr@live.com; 2 Psychology Department, Faculty of Educational Sciences and Psychology, Alzahra University, Tehran 1993893973, Iran; ab_mehrinejad@yahoo.com; 3 Faculty of Nursing and Midwifery, Kerman University of Medical Sciences, Kerman 7616913555, Iran; atefeahmadi59@gmail.com; 4 Kerman Nursing Research Center, Kerman University of Medical Sciences, Kerman 7616913555, Iran

**Keywords:** mindfulness, self-regulation, academic stress, undergraduate students

## Abstract

Academic stress is the most common emotional or mental state that students experience during their studies. Stress is a result of a wide range of issues, including test and exam burden, a demanding course, a different educational system, and thinking about future plans upon graduation. A sizeable body of literature in stress management research has found that self-regulation and being mindful will help students to cope up with the stress and dodge long-term negative consequences, such as substance abuse. The present study aims to investigate the influence of academic stress, self-regulation, and mindfulness among undergraduate students in Klang Valley, Malaysia, and to identify mindfulness as the mediator between academic stress and self-regulation. For this study, a total of 384 undergraduate students in Klang Valley, Malaysia were recruited. Using Correlational analysis, results revealed that there was a significant relationship between academic stress, self-regulation, and mindfulness. However, using SPSS mediational analysis, mindfulness did not prove the mediator role in the study.

## 1. Introduction

Today, almost everything is fast-paced, including technology, education, culture, and society. The high societal expectations on students to perform various undefined, inconsistent, and unachievable roles in the present socio-cultural, economic, and bureaucratic contexts of the society cause heavy stress on students [1]. Consequently, the current generation of students is much more stressed out and anxious when compared to previous generations [2,3]. This is because the current generation of students is seen as the most capable, especially with their proficiency in technology [4,5]; however, being seen as the capable ones have placed high expectations on them and they are being forced to follow unrealistic expectations [4]. Depression, anxiety, and stress have harmful effects on both individual and society. They can lead to negative outcomes, such as impaired normal functioning, burnout, and health problems. Undergraduate students in Malaysia are not excluded in this situation. 

Most Malaysian students suffer from excessive stress. One reason is the cultural pressure to graduate with good grades as graduating with good grades could give them an opportunity to find a better career [5]. These students generally have poor self-regulation and are unable to manage their stress well [2,6,7]. Malaysian students are unable to regulate themselves well when facing high levels of academic stress [8]. Moreover, undergraduate students in a public university in Malaysia have a low level of mindfulness [8]. 

To avoid such negative effects on students because of the stress, numerous approaches can be employed to overcome stress, such as self-regulation and mindful based intervention programs [9]. Self-regulation is defined as the ability to calm oneself when facing emotional discomfort and being able to cheer oneself up when experiencing dysphoria [10]. Individuals with ineffective self-regulation skills are at high risk of mental health disorders. Self-regulation abilities can predict individuals’ health problems, socioeconomic position, and tendency to criminal offence [11]. To achieve successful self-regulation, one must have both intrinsic and extrinsic motivation consistent with their goal of achieving behavior for a certain period [12]. Therefore, higher self-regulation is associated with high well-being, including good mental health, ability to maintain effective social relationships, and adaptive functioning in home or school [13]. According to [14], self-regulation abilities facilitate goal oriented actions and optimal adjusting to emotional and cognitive challenging stimulating throughout successful regulation of feelings, emotions, behaviours, and cognitions. 

In Malaysia, the major sources of stress among students are mostly difficulty in concentrating due to the presence of excessive information, the pressure of heavy workload, and examinations that cause harmful effects on their health and performance [15]. However, all of those academic life struggles can be overcome with using proper regulation. This statement is further supported by research on cognitive emotion regulation, as having positive reappraisal as part of coping strategy helps in overcoming academic life struggles [16].

In addition, the relationship between academic stress and self-regulation is supported by [17]. This study explored the differences in the self-regulation of stress management between students experiencing high stress levels and students experiencing low stress levels, among undergraduate students. This research found out that students with lower stress are better self-regulators. In addition, students with chronic stress frequently struggle to self-regulate caused by their inability to match their arousal state with their current circumstances [17,18].

Apart from self-regulation, mindfulness also plays an important role in our daily functioning. Mindfulness can be a helpful practice to handle the process of self-regulation [11,14,19]. A systematic review of mindfulness-based interventions in schools reported medium effect sizes on stress reduction and resilience [14]. Mindfulness in psychology means the state of being aware of the present feeling and the surrounding [20]. According to a definition by [19], mindfulness is “paying attention in a particular way, on purpose, in the present moment, and non-judgmentally”. Mindfulness involves acceptance, where we focus on our thoughts and feelings without having the sense of being judgmental [21]. Mindfulness plays a significant role in reducing stress, improving emotion-regulation, and developing greater awareness [22]. It is suggested that mindfulness training could help students to stay focused in class, because college is filled with distractions [23]. Students who tend to be more mindful than their peers are found to be non-judgmental and tend to act with awareness, being engaged in self-regulation [24]. Students with higher levels of mindfulness often have high levels of self-regulation [25,26]. This was further supported by another study that investigated the relationships between mindfulness, stress, coping styles, and substance abuse among university students, and concluded that mindfulness is an effective strategy for students to improve their coping strategy in reducing substance abuse caused by stress [27]. According to [27], mindfulness is also described as a cognitive style that facilitates boosting sense of mindscape awareness. They developed research on Mindfulness-Based Cognitive Therapy (MBCT) to help students in the Malaysian University to cope up with stress. In this study, they found out that MBCT has a significant reduction in perceived stress and an increase in mindfulness [28]. MBCT is a group based training programme developed based on Mindfulness-Based Stress Reduction Programme (MBSR). MBSR includes education about stress along with instruction on coping strategies. It involves the cultivation of attitudes, including becoming an impartial experience to one’s own knowledge and approval of things as they actually are at the moment. MBCT is based on MBSR and includes the principles and practices of cognitive therapy with mindfulness framework [29]. 

According to [30], the mindful coping process is a process of adjustment between adequate and changing inadequate coping responses. The process involves both problem-focused and emotion-focused strategies for an adequate coping in response to distress. When mindfulness is brought into this process, it may increase adequate coping. The process goes from mindfulness, bringing an increased consciousness of the situation, which is known as the primary appraisal. Then, once that is achieved, the process goes to the secondary appraisal, which is accepting awareness of the current situation and this acceptance provides access on how the individual would deal with the current stress. When dealing with the stress, it may involve both problem-focused coping, such as problem solving or emotion-focused coping; the reappraisal in this process that leads to positive adaptive coping. Tharaldsen mentioned that increased mindfulness through mindful-based training, can bring about mindfulness through associative processing and intuitive appraisal and when the individual face another distress, it will be easier for them to cope. When that happened, that person is known as a “successful coper”, an individual who is equipped with coping strategies and is flexible in adapting to their distressors [30].

Several studies are done in the mediating role of mindfulness. It is found that there is a negative relationship between trait mindfulness and perceived stress [31]. Those with higher trait mindfulness possess greater psychological flexibility and lower levels of psychological distress [9].

Furthermore, moderate levels of mindfulness is enough to regulate the perceived stress, as it is sufficient in raising an individual’s awareness based on their experience with stress [17]. A study on adults with problematic levels of stress revealed that mindfulness skills and perceived stress both change significantly from pretreatment to post-treatment [32]. They concluded that mindfulness changes the level of perceived stress; this finding is consistent with the previous studies that suggested improvements in mindfulness skills mediate the effects of mindfulness training on mental health outcomes. This statement is further supported by research conducted on mentally resilient athletes. The results showed that there are positive correlations between mindfulness and mental strength, proving that mindfulness is having a significant mediating effect on mental strength and athletic performance [33]. Based on the previous studies, there is a negative relationship between trait mindfulness and perceived stress [31]. As mentioned above, those with higher trait mindfulness possess greater psychological flexibility and lower levels of psychological distress [9]. Furthermore, there is evidence from [34] that mindfulness facilitate self-regulatory processes and coping, particularly during stressful experiences, as well as alleviating suffering among adolescents dealing with stress. Therefore, current study assumed that mindfulness acts as a mediator variable. However, mindfulness affects self-regulation significantly when it is accompanied with academic stress.

Concisely research on academic stress, self-regulation and mindfulness is not substantial in the Malaysian context. This study, therefore, serves as a direction to explore the relationship between academic stress, self-regulation, and mindfulness among undergraduate students in the Klang Valley, Malaysia. The findings of this research, may not be used to generalize the society globally, but only communities in the region, especially in Malaysia.

## 2. Materials and Methods

The research design for this research is quantitative and the data collected is through a survey. The samples are undergraduate students in Malaysia, specifically in the Klang Valley area. Using convenience sampling, the sample of 384 participants, the highest number of Krejcie & Morgan (1970) table [35], were recruited to achieve 95% confidence in generalizing an undisclosed total number of students in the Klang Valley, Malaysia. All of the participants included in the study were informed about confidentiality of the information provided. All participants provided with written consent and were informed that it was permissible to withdraw from participation in the study at any time. 

The study protocol was approved by the review board of psychology department, UCSI University to ensure ethical principles. Three instruments were used in this study. The first was The Perception of Academic Stress Scale (PAS) [36], which was used to measure the perception of academic stress among the undergraduate students. This instrument consisted of 18 items with an internal consistency of 0.70. PAS contains the academic expectations, faculty work, and examinations and students’ academic self-perceptions as its subscale. Internal consistency for each subscale was 0.72, 0.78, and 0.75, respectively. To measure self-regulation, The Self-Regulatory Inventory [37] was used. This instrument consisted of 36 items with an internal consistency of 0.822. This scale contains two subscales: short and long-term self-regulation with an internal consistency of 0.80 and 0.85, respectively. Finally, Mindful Attention Awareness Scale (MAAS), the trait version [38] was used to assess the participants’ mindfulness. This instrument is a one-dimensional scale, and it consists of 15 items with a high internal consistency of 0.90. The data collected was analysed with SPSS. To analyse the data, the Bivariate analysis was conducted using Pearson’s correlation. As for the mediation, it was tested using SPSS, mediational analysis developed by Preacher and Hayes. This analysis estimates direct and indirect effects in mediator models, interactions in moderation models [39].

## 3. Results

Table 1 shows the respondents’ demographic data. A total of 384 undergraduate students were involved in this study. Ninety-four were female respondents and 290 male respondents. The participants’ age was between 18 and 30, 334 in the age group of 22–25 years old and only 50 within 18–21 years old. Based on the descriptive statistics, 190 respondents were students from Social Sciences and Liberal Arts programme, 52 respondents from applied science programme, 37 respondents from Engineering programme, 10 respondents from Medical programme, 2 respondents from hospitality programme, and 25 respondents were from other programmes. While 250 respondents were from private universities, 134 respondents were from public universities in the Klang Valley, Malaysia.

According to Table 2, faculty work and examination caused undergraduate students’ academic stress, as it shows higher mean for both age groups (*M* = 26.66) for 18–20 and (*M* = 25.61) for 21–30, as compared to academic self-perception (*M* = 19.86) for 18–20 and (*M* = 21.07) for 21–30 and academic expectation (*M* = 11.94) for 18–20 and (*M* = 12.41) for 21–30. As for self-regulation, the undergraduate students from both age groups appeared to score higher in long-term self-regulation. This means that they have the greater ability to self-regulate in the long-term. Finally, for mindfulness, they appeared to have a moderate level of mindfulness (*M* = 55.92) for 18–20 and (*M* = 54.39) for 21–30 as the highest score for mindfulness is 88.

To investigate the intercorrelations of academic stress, self-regulation, and mindfulness, the Pearson correlation was used. From Table 3, the relationship between self-regulation (*r* = −0.165, *p* < 0.05) and mindfulness (*r* = −0.109, *p* < 0.05) was significantly and negatively correlated with academic stress. The third relationship between mindfulness and self-regulation (*r* = 0.043, *p* < 0.05) was significantly correlated. The results indicates the higher academic stress among student, the lower self-regulation, and mindfulness, and the higher mindfulness, the higher self-regulation that they possess.

**Figure 1:**
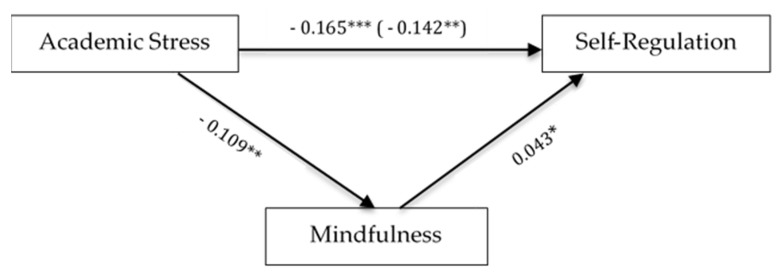
Mediator Effect of Mindfulness. Note: * *p* < 0.05; ** *p* < 0.01; *** *p* < 0.001.

Figure 1 illustrates the mediation model of mindfulness on the relationship between academic stress and self-regulation using SPSS PROCESS macro by Preacher and Hayes [40]. In step 1 of the mediation model, the regression of academic stress on self-regulation, ignoring the mediator, was significant, β = −0.165, *t* = −3.255, *p* < 0.001. Step 2 showed that the regression of academic stress on mediator, mindfulness, was also significant, β = −0.109, *t* = 149.2, *p* < 0.01. Step 3 showed that the regression of mindfulness on self-regulation, controlling for academic stress, was significant, β = 0.043, *t* = 838.0, *p* < 0.05. Lastly, Step 4, revealed that academic stress predicted self-regulation, while controlling for mindfulness, β = −0.142, *t* = 781.2, *p* < 0.01. The Sobel test of the indirect effect was not significant, indicating no mediation (Z = 0.4741, *p* > 0.05). Using 10,000 boot-strapped samples, the estimate of the indirect effect again suggested no mediation, with a point estimate of 0.005 (SE = 0.012, 95% CI = −0.119 to 0.038). It can be concluded that mindfulness does not mediate the relationship between academic stress and self-regulation. In addition, sub-types of self-regulation were considered in the model and mediation effect of mindfulness was tested. The Sobel test of the indirect effect showed no mediation effect for both sub-types of self-regulation. For short term self-regulation, the Sobel test was not significant (Z = 0.193, *p* > 0.05), as using 10,000 boot-strapped samples, the estimate of the indirect effect suggested no mediation, with a point estimate of 0.000 (SE = 0.004, 95% CI = −0.008 to 0.011). For long term self-regulation, the Sobel test also was not significant (Z = 0.434, *p* > 0.05), using 10,000 bootstrapped samples, the estimate of the indirect effect suggested no mediation, with a point estimate of 0.002 (SE = 0.006, 95% CI = 0.000 to 0.003).

## 4. Discussion

This study investigated the intercorrelations of academic stress, self-regulation, and mindfulness among undergraduate students in Malaysia. The results showed that there is a significant negative relationship between academic stress and self-regulation among undergraduate students in Malaysia. This corroborates with the previous research, which indicated that high levels of stress are negatively associated with executive functioning, such as self-regulation [40]. Another finding demonstrated that higher levels of perceived stress restricted the students’ ability to utilize executive functioning skills, such as critical thinking strategies for complex action and overrides emotional responses from engaging in goal-directed behavior [41]. In addition, a significant negative correlation was found between academic stress and mindfulness among undergraduate students in Malaysia. The findings of this study showed that students, when facing academic stress, have low levels of mindfulness. This supports a previous research on investigating how students’ practices on mindful attention are associated with their level of mindlessness and stress during undergraduate studies [42], which states that a lower level of mindful attention is often associated with carelessness, inattention, high level of stress, and overall lower well-being. This is because when a situation is regarded as stressful, there is a high chance that an unmindful person would unconsciously enact avoidance behaviors, such as procrastination or emotion-focused behavior, such as substance abuse [43,44]. Another finding indicated that higher levels of perceived stress restricted the student’s ability to utilize executive functioning skills, such as critical thinking strategies for complex action and this, overrides their emotional responses from engaging in goal-directed behavior [41].

Furthermore, the results indicated that there is a significant relationship between mindfulness and self-regulation among undergraduate students in Malaysia. This finding is supported by previous research, which indicated that students who have higher levels of mindfulness often have a high level of self-regulation [25]. An individual who approaches situations with mindfulness is more likely to act in accordance with their values and beliefs [38]. This is because the individual learns that such situations are manageable, and it does not necessitate the emotion-focused coping strategy [45]. 

As for the mediation analysis, the results showed that there is no mediating effect of mindfulness on academic stress and self-regulation. This research finding is in contrast with previous research on undergraduate students in investigating the relationships among mindfulness, self-regulated learning (SRL), and perceived stress. It states that mindfulness partially mediates the relation between perceived stress and self-regulated learning [26]. Although this study has concluded that mindfulness does not mediate the role on academic stress and self-regulation, but mindfulness somehow has a significant relationship with academic stress and self-regulation, based on the correlational analysis. Therefore, the importance of mindfulness in daily life, especially in managing stress, cannot be neglected. Mindfulness is not widely practised among undergraduate students in Malaysia. This can be seen from a study that was done on medical students in the Malaysian University. The results showed that only after they participated in Mindfulness-Based Cognitive therapy, they display a reduction in perceived stress [46].

It is believed that the implementation of mindfulness practices in classrooms will help to promote mindfulness and may help the student to reduce their stress levels. Practicing mindfulness may assist the student in their self-regulation. The faculty work and examinations that are the main causes of students’ academic stress based on this study are somehow manageable by practising mindfulness and self-regulation as their coping strategy. Therefore, students can benefit from mindfulness practices to control their negative emotions, thoughts, and behaviors throughout university life, and continue to excel in their academic life. 

According to the reviewed literature, most studies in mindfulness have focused on Mindfulness-Based Cognitive Therapy (MBCT) and Mindful-Based Stress Reduction Training (MBSR). There is still lack of research on mindfulness itself, since the role of mindfulness is still very unclear to individuals. The future researchers who intend to study mindfulness further could explain the role of mindfulness in their basic executive functioning, such as working memory. Therefore, in-depth research on the role of mindfulness, especially in academic stress and self-regulation, is very much needed.

As for the self-regulation, it is recommended that the future researchers focus on specific aspects of self-regulation, such as impulsive control, rather than attempting to study self-regulation in its broader sense.

## Figures and Tables

**Table 1:** Frequency and percentage of respondents’ demographic data (N = 384).

Demographic	Frequency (N = 384)	Percentage (%)
**Gender**		
Female	290	75.5
Male	94	24.5
**Ethnicity**		
Malay	117	46.1
Chinese	134	34.9
Indian	37	9.6
Other	36	9.4
**Age**		
18–21	50	13.0
22–25	334	87.0
**Programme**		
Social Sciences and Liberal Arts	190	49.5
Applied Science	52	13.5
Business and Information Science	68	17.7
Engineering, Technology and Built Environment	37	9.6
Medicine and Health Sciences	10	2.6
Hospitality and Health Management	2	0.5
Others	25	6.5
**Type of Institution**		
Private	250	65.1
Public	134	34.9

**Table 2:** Mean and Standard Deviation of Subscales for Age Groups.

Subscales		Age Group 18–20		Age Group 21–30	
		Mean	SD	Mean	SD
**Academic Stress**	Academic Expectation	11.94	3.106	12.41	3.016
	Faculty work and Examination	26.66	4.872	25.60	4.316
	Academic Self-Perception	19.86	3.539	21.06	3.332
**Self-Regulation**	Short-Term Effect	41.56	4.669	44.37	6.098
	Long-Term Effect	43.38	8.909	47.99	8.121
**Mindfulness**		55.92	11.463	54.39	8.425

**Table 3:** Pearson’s correlation between variables (N = 384).

Variables	r	Sig. (2-Tailed)
Academic Stress Self-Regulation	−0.165 **	0.004
Academic Stress Mindfulness	−0.109 *	0.032
Mindfulness Self-Regulation	0.043 *	0.015

Note: * *p* < 0.05; ** *p* < 0.01.

## References

[1] Lal K. (2014). Academic Stress among Adolescent in Relation to Intelligence and Demographic Factors. Am. Int. J. Res. Humanit. Arts Soc. Sci..

[2] Teh C., Ngo C., Zulkifli R., Vellasamy R., Suresh K. (2015). Depression, Anxiety and Stress among Undergraduate Students: A Cross Sectional Study. Open J. Epidemiol..

[3] Leppink E., Odlaug B., Lust K., Christenson G., Grant J. (2016). The Young and the Stressed. J. Nerv. Ment. Dis..

[4] Howe N., Strauss W. (2007). Millennials Go to College.

[5] The Coverage Bureau (2015). Malaysia Sunway University Student Tragically Commits Suicide. http://thecoverage.my/youth/malaysia-sunway-university-student-tragically-commits-suicide/.

[6] Faleel S.F., Tam C.L., Lee T.H., Har W.M., Foo Y.C. (2012). Stress, perceived social support, coping capability and depression: A study of local and foreign students in the Malaysian context. Int. J. Soc. Hum. Sci..

[7] Radeef A., Faisal G., Ali S., Ismail M. (2014). Source of stressors and emotional disturbances among undergraduate science students in Malaysia. Int. J. Med. Res. Health Sci..

[8] Ahmadi A., Mustaffa M., Haghdoost A., Alavi M. (2014). Mindfulness and Related Factors among Undergraduate Students. Procedia Soc. Behav. Sci..

[9] Bamber M.D., Schneider J.K. (2016). Mindfulness-based Meditation to Decrease Stress and Anxiety in College Students: A Narrative Synthesis of the Research. Educ. Res. Rev..

[10] McClelland M., Geldhof J., Morrison F., Gestsdóttir S., Cameron C., Bowers E., Duckworth A., Little T., Grammer J., Halfon N., Forrest C.B., Lerner R.M., Faustman E.M. (2018). Self-Regulation. Handbook of Life Course Health Development.

[11] Kaunhoven R.J., Dorjee D. (2017). How does mindfulness modulate self-regulation in pre-adolescent children? An integrative neurocognitive review. Neurosci. Biobehav. Rev..

[12] Berkman E. (2016). Handbook of Self-Regulation.

[13] Buckner J.C., Mezzacappa E., Beardslee W.R. (2009). Self-Regulation and its Relations to Adaptive Functioning in Low Income Youths. Am. J. Orthopsychiatr..

[14] Zenner C., Herrnleben-Kurz S., Walach H. (2014). Mindfulness-based interventions in schools-A systematic review and meta-analysis. Front. Psychol..

[15] Poon W., Lee C., Ong T. (2012). Undergraduates’ perception on causes, coping and outcomes of academic stress: Its foresight implications to university administration. Int. J. Foresight Innov. Policy.

[16] Panahi S., Yunus A., Panahi M. Influence of Cognitive Emotion Regulation on Psychological Well-being of Malaysian Graduates. Proceedings of the 4th International Congress on Clinical and Counselling Psychology.

[17] Winterbach L. (2007). Self- Regulation and Stress Management in Undergraduate Students. Master’s Thesis.

[18] Tranter D., Kerr D. (2016). Understanding Self-Regulation: Why Stressed Students Struggle to Learn (1st ed.). http://www.edu.gov.on.ca/eng/literacynumeracy/inspire/research/ww_struggle.

[19] Kabat-Zinn J. (2003). Mindfulness-based interventions in context: Past, present, and future. Clin. Psychol. Sci. Pract..

[20] Davis D., Hayes J. (2011). What Are the Benefits of Mindfulness? A Practice Review of Psychotherapy-related Research. Psychotherapy.

[21] Zoogman S., Goldberg S.B., Hoyt W.T., Miller L. (2015). Mindfulness interventions with youth: A meta-analysis. Mindfulness.

[22] Brausch D. (2011). The Role of Mindfulness in Academic Stress, Self-Efficacy, and Achievement in College Students. Master’s Thesis.

[23] Caldwell K., Harrison M., Adams M., Quin R.H., Greeson J. (2010). Developing Mindfulness in College Students through Movement-Based Courses: Effects on Self-Regulatory Self-Efficacy, Mood, Stress, and Sleep Quality. J. Am. Coll. Health.

[24] Short M., Mazmanian D., Oinonen K., Mushquash C. (2016). Executive Function and Self-Regulation Mediate Dispositional Mindfulness and Well-Being. Personal. Individ. Differ..

[25] Jimenez S., Niles B., Park C. (2010). A Mindfulness Model of Affect Regulation and Depressive Symptoms: Positive Emotions, Mood Regulation Expectancies, and Self-Acceptance as Regulatory Mechanisms. Personal. Individ. Differ..

[26] Trevisani C. (2015). A Correlational Study of Self-Regulated Learning, Stress and Mindfulness in Undergraduate Students. Undergraduate Thesis.

[27] Thomas C. (2011). A Review of the Relationships between Mindfulness, Stress, Coping Styles and Substance Use among University Students. Undergraduate Thesis.

[28] Kar P., Ling K., Chong C. (2014). Mindful-S.T.O.P.: Mindfulness Made Easy for Stress Reduction in Medical Students. Educ. Med. J..

[29] Segal Z.V., Williams J.M.G., Teasdale J.D. (2012). Mindfulness-Based Cognitive Therapy for Depression.

[30] Tharaldsen K.B. (2012). Mindful Coping. Ph.D. Thesis.

[31] Weinstein N., Brown K., Ryan R. (2009). A multi-method examination of the effects of mindfulness on stress attribution, coping, and emotional well-being. J. Res. Personal..

[32] Baer R., Carmody J., Hunsinger M. (2012). Weekly Change in Mindfulness and Perceived Stress in a Mindfulness-Based Stress Reduction Program. J. Clin. Psychol..

[33] Abdul Rafeeque T.C., Sultana D. (2016). Mediating Role of Mindfulness on the Relationship between Mental Toughness and Athletics Performance of Inter University Track and Field Athletes (1st ed.). Int. J. Phys. Educ. Sports Health.

[34] Perry-Parrish C., Copeland-Linder N., Webb L., Shields A.H., Sibinga E.M. (2016). Improving self-regulation in adolescents: Current evidence for the role of mindfulness-based cognitive therapy. Adolesc. Health Med. Ther..

[35] Krejcie R.V., Morgan D.W. (1970). Determining Sample Size for Research Activities. Educ. Psychol. Meas..

[36] Bedewy D., Gabriel A. (2015). Examining Perceptions of Academic Stress and Its Sources among University Students: The Perception of Academic Stress Scale. Health Psychol. Open.

[37] Moilanen K. (2007). The Adolescent Self-Regulatory Inventory: The Development and Validation of a Questionnaire of Short-Term and Long-Term Self-Regulation. J. Youth Adolesc..

[38] Brown K., Ryan R. (2003). The benefits of being present: Mindfulness and its role in psychological well-being. J. Personal. Soc. Psychol..

[39] Preacher K.J., Hayes A.F. (2004). SPSS and SAS Procedures for Estimating Indirect Effects in Simple Mediation Models. Behav. Res. Methods Instrum. Comput..

[40] Orem D.M., Petrac D.C., Bedwell J.S. (2008). Chronic self—Perceived stress and set—Shifting performance in undergraduate students. Stress Int. J. Biol. Stress.

[41] Williams P., Suchy Y., Rau H. (2009). Individual Differences in Executive Functioning: Implications for Stress Regulation. Ann. Behav. Med..

[42] Bahl S., Milne G., Ross S., Chan K. (2013). Mindfulness: A Long-Term Solution for Mindless Eating by College Students. J. Public Policy Mark..

[43] Breslin F.C., Zack M., McCain S. (2002). An Information-Processing Analysis of Mindfulness: Implications for Relapse Prevention in the Treatment of Substance Abuse. Clin. Psychol. Sci. Pract..

[44] Shapiro S., Carlson L., Astin J., Freedman B. (2006). Mechanisms of Mindfulness. J. Clin. Psychol..

[45] Carmody J. (2009). Evolving Conceptions of Mindfulness in Clinical Settings. J. Cogn. Psychother..

[46] Phang C., Chiang K., Ng L., Keng S., Oei T. (2015). Effects of Brief Group Mindfulness-based Cognitive Therapy for Stress Reduction among Medical Students in a Malaysian University. Mindfulness.

